# The effects of graded motor imagery and its components on phantom limb pain and disability in upper and lower limb amputees: a systematic review protocol

**DOI:** 10.1186/s13643-016-0322-5

**Published:** 2016-09-01

**Authors:** Katleho Limakatso, Lieselotte Corten, Romy Parker

**Affiliations:** 1 Division of Physiotherapy, Department of Health and Rehabilitation Sciences, University of Cape Town, Cape Town, South Africa; 2 Division of Physiotherapy, Department of Health and Rehabilitation Sciences, Groote Schuur Hospital, University of Cape Town, F45 Old Main Building, Cape Town, South Africa

**Keywords:** Phantom limb pain, Disability, Graded motor imagery, Laterality recognition, Explicit motor imagery, Mirror visual feedback

## Abstract

**Background:**

Phantom limb pain (PLP) is characterized by the anatomical shifting of neighbouring somatosensory and motor areas into a deafferented cortical area of the brain contralateral to the amputated limb. It has been shown that maladaptive neuroplasticity is positively correlated to the perception of PLP in amputees. Recent studies support the use of graded motor imagery (GMI) and its component to alleviate the severity of PLP and disability. However, there is insufficient collective empirical evidence exploring the effectiveness of these treatment modalities in amputees with PLP. This systematic review will therefore explore the effects of GMI and its individual components on PLP and disability in upper and lower limb amputees.

**Methods:**

We will utilize a customized search strategy to search PubMed, Cochrane Central register of Controlled Trials, MEDLINE, Embase, PsycINFO, PEDro, Scopus, CINAHL, LILACS, DARE, Africa-Wide Information and Web of Science. We will also look at clinicaltrials.gov (http://www.clinicaltrials.gov/), Pactr.gov (http://www.pactr.org/) and EU Clinical trials register (https://www.clinicaltrialsregister.eu/) for ongoing research. Two independent reviewers will screen articles for methodological validity. Thereafter, data from included studies will be extracted by two independent reviewers through a customized pre-set data extraction sheet. Studies with a comparable intervention and outcome measure will be pooled for meta-analysis. Studies with high heterogeneity will be analysed through random effects model. A narrative data analysis will be considered where there is insufficient data to perform a meta-analysis.

**Discussion:**

Several studies investigating the effectiveness of GMI and its different components on PLP have drawn contrasting conclusions regarding the efficacy and applicability of GMI in clinical practice. This systematic review will therefore gather and critically appraise all relevant data, to generate a substantial conclusion and recommendations for clinical practice and research on this subject.

**Systematic review registration:**

PROSPERO CRD42016036471

**Electronic supplementary material:**

The online version of this article (doi:10.1186/s13643-016-0322-5) contains supplementary material, which is available to authorized users.

## Background

### Description of condition

Amputation is the removal of a body extremity which is generally caused by severe trauma, circulatory disorders, neoplasm, deformities and infection of the limb. In case of gangrene, infection or neoplasm, amputation is carried out as a control strategy for pre-operative pain or a disease process in the affected limb; in some cases, however, amputation surgery is performed as a preventative procedure for the abovementioned complications [31]. Despite this attempt to alleviate patients’ pain and disability, up to 80 % of amputees report phantom limb pain (PLP) post-amputation surgery [8]. PLP is defined as persistent painful sensations perceived in the missing portion of the amputated limb [7]. Previous research associates PLP with peripheral changes such as increased nociceptive input from the residual limb [39] and reduced near-surface blood-flow [34]. However, recent evidence suggests that PLP is a sensory output primarily driven by cortical changes in the brain [10]. Neuroimaging studies of patients with PLP revealed neuroplastic alterations of the somatotopic organization of the cortical and sub-cortical areas of the brain [9, 13]. These changes are characterized by the anatomical shifting of neighbouring somatosensory [18] and motor [4] areas into a deafferented cortical area of the brain contralateral to the amputated limb. Furthermore, these neuroplastic changes are positively correlated to the severity of PLP [15, 17]. These neuroplastic alterations can be reverted, with a correlation between the reversal of neuroplastic changes and pain relief in amputees with PLP [1, 11, 20].

### Description of intervention

Graded motor imagery (GMI) is a treatment strategy which has been shown to mitigate the severity of PLP and disability using a sequence of strategies including laterality recognition, explicit motor imagery and mirror visual feedback [22].

Laterality recognition, the ability to distinguish left from right, is dependent on the intact body schema in the brain and is important in the planning of movement [32]. This left/right judgement is inaccurate and delayed in amputees with PLP [25]. In this first phase of GMI, images representing the amputated limb are presented randomly on a computer screen. The patient is then instructed to match the side of the presented limbs by pressing either the left or right key. During this task, emphasis is put on accuracy and speed.

Laterality recognition is alternately known as implicit motor imagery, primarily because the patient is unconscious of mental movement processes involved to match the limb presented on the computer screen [5].

During the explicit motor imagery phase of GMI, the patient mentally moves the amputated limb to adopt a desirable posture presented on the computer screen [14].

The last strategy of GMI is mirror visual feedback, during which the amputated limb is concealed behind a mirror with the intact limb positioned comfortably in front of the mirror. This superimposes the ocular image of the intact limb on the phantom limb [28]. The patient is shown a picture of the unaffected limb in an easy to attain position. The patient then simultaneously moves the intact limb and the phantom limb (through imagination) to the presented position while observing the reflection of the intact limb in the mirror. This phase can commence with gross movements, progress to fine motor tasks and ultimately functional tasks.

### How the intervention might work

GMI is consistent with sequential activation of cortical premotor and motor networks [23]. The GMI intervention is founded on the principle of graded increase in activity, similar to that implemented in physiotherapy treatment modalities [2]. This graded exposure to activity aims to promote cortical re-organization without triggering the protective pain response.

Laterality recognition/implicit motor imagery tasks activate premotor and supplementary motor areas, with an exception of the primary motor (M1) cortex [24]. Laterality recognition is therefore fundamental in preparation for subsequent phases of the GMI programme.

Explicit motor imagery activates the somatosensory (S1), premotor and M1 cortices contralateral to the phantom limb [17]. Activation of these areas is proposed to alleviate the perception of pain associated with imagination and observation of movement. This phase builds upon laterality recognition and serves as a foundation for the successive GMI phase.

Mirror visual feedback addresses changes in the S1 and M1 cortices [26]. In addition, it provides visual input to the brain, that movement is executed normally without inhibition [21]. The therapeutic effect associated with mirror visual feedback may be due to activation of mirror neurons in the brain hemisphere contralateral to the amputated limb [3]. These mirror neurons have been shown to fire during observation and execution of movement [16, 30].

### Importance of doing this review

Several studies [3, 19, 20, 22, 36, 38], investigating the effectiveness of GMI and its different components on PLP, have drawn contrasting conclusions regarding the efficacy and applicability of GMI in clinical practice.

This systematic review will therefore gather and critically appraise all relevant data, to generate a substantial conclusion and recommendations for clinical practice and research on this subject.

It is important to note that Plumbe and associates have published a protocol with a similar topic. Despite this similarity, there is a significant distinction between the two protocols. In their review, Plumbe et al. [27] indicate that they will investigate the efficacy of GMI on chronic pain. However, this review will explore the effects of GMI and its components, specifically on PLP and disability. Bowering et al. [2] conducted a systematic review on a topic of interest. However, there is a need to update their review as there have been new studies since their publication.

## Objectives

The purpose of this review is to explore the effects of GMI and its individual components on PLP and disability in upper and lower limb amputees.

## Methods

The protocol was developed according to the Preferred Reporting Items of Systematic Reviews and Meta-Analyses Protocol (PRISMA-P) guidelines [33] and has been registered on PROSPERO database (Ref: CRD42016036471). The PRISMA-P checklist is included as an additional file (Additional file 1).

## Criteria for selecting studies for this review

### Types of studies

This systematic review will consider published and non-published randomized controlled trials, quasi-experimental studies, randomized controlled cross-over trials and quasi-experimental cross-over studies published in English.

### Types of participants

Participants older than 18 years of age with unilateral amputation of the upper or lower limb who received intervention for their PLP three or more months post-amputation surgery will be included in this review. Studies with participants with pathology of the intact opposite limb will be excluded.

### Types of interventions

GMI and its individual components (laterality recognition, explicit motor imagery and mirror visual feedback) will be compared to no treatment, conventional physiotherapy or other interventions. Studies investigating the efficacy of GMI plus additional treatment will also be included.

### Conventional physiotherapy includes:

- Transcutaneous electrical nerve stimulation
- Muscle relaxation/massage
- Heat/cryotherapy
- Acupuncture
- Exercise
- Biofeedback

Other interventions include:

- Pharmacological interventions
- Psychotherapy
- Deep brain stimulation
- Motor cortex stimulation
- Spinal cord stimulation

### Types of outcome measures

Primary outcome measures

- Self-reported PLP as assessed through a standardized pain scale post-intervention
- Pain-related disability as assessed through a standardized function scale post-treatment

Secondary outcome measures:

- Health-related quality of life (HRQoL) as assessed by a standardized scale
- Adverse effects
- Psychosocial function as assessed by a standardized scale
- Patient global impressions of change (PGIC) after 3 months or more

## Search strategy for identification of studies

### Electronic searches

We will utilize a customized search strategy (Appendix) to search the following electronic databases: PubMed, Cochrane Central register of Controlled Trials, MEDLINE (via Ebscohost), Embase, PsycINFO (via Ebscohost), Physiotherapy Evidence Database (PEDro), Scopus, Cumulative Index to Nursing and Allied Health Literature (CINAHL) (via Ebscohost), LILACS, Database of Abstracts of Reviews of effects in the Cochrane Library (DARE) Africa-Wide Information (via Ebscohost) and Web of Science. We will also look at clinicaltrials.gov (http://www.clinicaltrials.gov/), Pactr.gov (http://www.pactr.org/) and EU Clinical trials register (https://www.clinicaltrialsregister.eu/) for ongoing research.

### Search of other sources

The reference lists of identified studies will be searched for relevant additional trials. Experts in this field will be contacted for further identification of published, unpublished and ongoing studies with potential for inclusion in this review.

## Data collection and analysis

### Selection of studies

Databases will be searched by one reviewer to identify potential titles and abstracts. Two reviewers will independently screen these titles and abstracts for methodological validity. Full articles of relevant studies will be obtained and scrutinized by two independent reviewers to determine eligibility for inclusion in this review. Should there be a disagreement between reviewers, a consensus will be reached through discussion. However, should this fail, a third reviewer will be requested to take a decision.

### Data extraction and management

Data from included studies will be extracted by two independent reviewers through a customized pre-set data extraction sheet. Extracted data will include the following: country of origin, study design (parallel/cross-over/cluster, randomisation, allocation concealment and blinding) and professional discipline of clinician delivering the intervention; setting, number of participants per group and points estimates; comorbidities, exclusion/inclusion criteria, participants’ age and gender; type and side of amputation, adverse effects, pain condition and period (months) post-amputation; assessment tools, type of treatment and control intervention received; duration of treatment (minutes), frequency of treatment per week, follow-up period (weeks) and number of patients lost to follow-up; baseline, post-intervention and follow-up results on outcome measures and author conflict of interest statement. Data will be recorded into Review Manager 5 [37]. Disagreements concerning data extraction will be resolved through discussion. A third reviewer will be consulted where a consensus cannot be reached.

### Assessment of risk of bias

Two independent reviewers will utilize the Cochrane method for risk of bias assessment. Included studies will be classified as low, high or unclear risk of bias. Any disagreements between reviewers will be resolved through discussion. A third reviewer will be consulted where a consensus cannot be reached. We will acknowledge and report on concerns of bias that can influence the outcome of this review, in particular selection bias, performance bias and publication bias

### Measures of treatment effect

For studies with a comparable outcome measure, we will pool data for meta-analysis. Continuous data will be presented as 95 % confidence interval (CI) and mean difference (MD). The risk ratio (RR) and 95 % CI will be calculated for dichotomous data. Furthermore, the number needed to treat (NNT) will be calculated. According to Initiative on Methods, Measurement, and Pain Assessment in Clinical Trials (IMMPACT), ≥15 % pain reduction is minimally significant, ≥30 % pain reduction is moderately significant and ≥50 % is considerably significant [6].

### Unit of analysis issues

For cross-over experimental design studies, only the first set of data will be considered for analysis. When trials have more than one intervention group, the control group will be divided in half. Each group will separately be included in the meta-analysis [29].

We will classify follow-up outcome measures by duration; short-term (up to 3 months post-treatment), medium-term (3–9 months post-treatment) and long-term (longer than 2 years post-treatment).

### Dealing with missing data

In case of missing data, we will contact authors to provide further information. When authors fail to provide information within 2 months, we will use intention-to-treat analysis for extrapolated data. The impact of this will be reported in the discussion section of the systematic review.

### Assessment of heterogeneity

Clinical heterogeneity will be addressed by pooling studies which investigate the same intervention and outcomes in amputees with PLP. Methodological heterogeneity will be examined visually, whereas statistical heterogeneity will be assessed through *I*^2^ statistics. We will consider a cut-off score of 50 %.

### Assessment of reporting biases

We will compare the methods section of the articles with the results section. If sufficient data is included, we will assess reporting bias by using a funnel plot [35].

### Data synthesis

Studies with a comparable intervention and outcome measure will be pooled for meta-analysis using Review Manager 5 [37]. We will use the random effects model to analyse studies with high heterogeneity. We will consider narrative data analysis where there is insufficient data to perform a meta-analysis.

### Sensitivity analysis:

If numerous trials satisfy the inclusion criteria for this systematic review, sensitivity analysis will be conducted to examine the possibility of excluding studies with a high risk of bias.

### Subgroup analysis

A subgroup analysis depending on age, gender or type of amputee will be performed when applicable.

### Grading the certainty of evidence

We will grade the certainty of the evidence for all outcome measures using the Grading of Recommendations Assessment, Development, and Evaluation working group methodology [12]

## Discussion

Given the prevalence of amputees with PLP and disability, it is imperative to generate a substantial conclusion regarding the application of motor imagery techniques in clinical practice. The proposed systematic review will therefore explore the efficacy of GMI and its components as treatment modalities for PLP and disability. GMI is a cost-effective and non-invasive treatment with limited adverse effects and complications. As such, significant outcomes from this review will guide patients and clinicians in their treatment selection. Furthermore, these significant findings will reinforce the implementation of GMI in clinical practice, therefore making it accessible to low income upper and lower limb amputees with PLP and disability.

## Appendix

Search strategy

1. (graded motor imagery OR motor imagery programme OR movement representation techniques)
2. (laterality recognition OR left right judge* OR implicit motor imagery)
3. (imagined movement OR explicit motor imagery OR mental imagery)
4. (mirror visual feedback OR mirror therap* OR mirror technique* OR mirror box therap*)
5. (phantom limb pain OR phantom pain)
6. (amput*)
7. 1 OR 2 OR 3 OR 4
8. 5 AND 6
9. 7 AND 8

## Additional file

Additional file 1: PRISMA-P (Preferred Reporting Items for Systematic review and Meta-Analysis Protocols) 2015 checklist: recommended items to address in a systematic review protocol*.

## Abbreviations

PLP: Phantom limb pain
GMI: Graded motor imagery
M1: Primary motor
S1: Somatosensory
PRISMA-P: Preferred Reporting Items of Systematic Reviews and Meta-Analyses Protocol
HRQoL: Health-related quality of life
PGIC: Patient global impressions of change
PEDro: Physiotherapy Evidence Database
CINAHL: Cumulative Index to Nursing and Allied Health Literature
DARE: Database of Abstracts of Reviews of Effects
CI: Confidence interval
MD: Mean difference
RR: Risk ratio
NNT: Number needed to treat
IMMPACT: Initiative on Methods, Measurement, and Pain Assessment in Clinical Trials
