# Supplementary material for: The effects of graded motor imagery and its components on phantom limb pain and disability in upper and lower limb amputees: a systematic review protocol
Source: Syst Rev. 2016 Sep 1;5(1):145. doi: 10.1186/s13643-016-0322-5 (PMC5007706; doi:10.1186/s13643-016-0322-5)
Supplement: Additional file 1: — PRISMA-P (Preferred Reporting Items for Systematic review and Meta-Analysis Protocols) 2015 checklist: recommended items to address in a systematic review protocol*. [file 13643_2016_322_MOESM1_ESM.docx]

# Additional File 1:

**PRISMA-P (Preferred Reporting Items for Systematic review and Meta-Analysis Protocols) 2015 checklist: recommended items to address in a systematic review protocol***

| **Section and topic** | **Item No** | **Checklist item** |
| --- | --- | --- |
| **ADMINISTRATIVE INFORMATION** | | |
| Title: |  |  |
| Identification | 1a | Identify the report as a protocol of a systematic review  Yes- ***The effects of Graded Motor Imagery and its components on phantom limb pain and disability in upper and lower limb amputees: A systematic review protocol.*** |
| Update | 1b | If the protocol is for an update of a previous systematic review, identify as such  N/A |
| Registration | 2 | If registered, provide the name of the registry (such as PROSPERO) and registration number  PROSPERO CRD42016036471 |
| Authors: |  |  |
| Contact | 3a | Provide name, institutional affiliation, e-mail address of all protocol authors; provide physical mailing address of corresponding author  Yes- please refer to Title page |
| Contributions | 3b | Describe contributions of protocol authors and identify the guarantor of the review  Yes- please refer to “Authors’ contribution” |
| Amendments | 4 | If the protocol represents an amendment of a previously completed or published protocol, identify as such and list changes; otherwise, state plan for documenting important protocol amendments  N/A |
| Support: |  |  |
| Sources | 5a | Indicate sources of financial or other support for the review  N/A |
| Sponsor | 5b | Provide name for the review funder and/or sponsor  Oppenheimer Memorial Trust |
| Role of sponsor or funder | 5c | Describe roles of funder(s), sponsor(s), and/or institution(s), if any, in developing the protocol  N/A |
| **INTRODUCTION** | | |
| Rationale | 6 | Describe the rationale for the review in the context of what is already known  Yes- “Several studies (Ulger et al., 2009; Maclver et al., 2008; Sumitani et al., 2008; Chan et al., 2007; Moseley, 2006 & MacLachlan, 2004), investigating the effectiveness of GMI and its different components on PLP, have drawn contrasting conclusions regarding the efficacy and applicability of GMI in clinical practice.  This systematic review will therefore gather and critically appraise all relevant data, to generate a substantial conclusion and recommendations for clinical practice and research on this subject. ” |
| Objectives | 7 | Provide an explicit statement of the question(s) the review will address with reference to participants, interventions, comparators, and outcomes (PICO)  Yes- “The purpose of this review is to explore the effects of GMI and its individual components on PLP and disability in upper and lower limb amputees.  ” |
| **METHODS** | | |
| Eligibility criteria | 8 | Specify the study characteristics (such as PICO, study design, setting, time frame) and report characteristics (such as years considered, language, publication status) to be used as criteria for eligibility for the review  Yes- ,our eligibility criteria considers population, intervention, outcomes assessed and study characteristics |
| Information sources | 9 | Describe all intended information sources (such as electronic databases, contact with study authors, trial registers or other grey literature sources) with planned dates of coverage  Yes- this include electronic databases and contacting authors |
| Search strategy | 10 | Present draft of search strategy to be used for at least one electronic database, including planned limits, such that it could be repeated  Yes- please refer to Appendix 1 |
| Study records: |  |  |
| Data management | 11a | Describe the mechanism(s) that will be used to manage records and data throughout the review  Yes- Data will be recorded into Review Manager 5 |
| Selection process | 11b | State the process that will be used for selecting studies (such as two independent reviewers) through each phase of the review (that is, screening, eligibility and inclusion in meta-analysis)  Yes- “Databases will be searched by one reviewer to identify potential titles and abstracts. Two reviewers will independently screen these titles and abstracts for methodological validity. Full articles of relevant studies will be obtained and scrutinized by two independent reviewers to determine eligibility for inclusion in this review. Should there be a disagreement between reviewers, a consensus will be reached through discussion. However, should this fail, a third reviewer will be requested to take a decision.” |
| Data collection process | 11c | Describe planned method of extracting data from reports (such as piloting forms, done independently, in duplicate), any processes for obtaining and confirming data from investigators  Yes- “Data from included studies will be extracted by two independent reviewers through a customised pre-set data extraction sheet. Disagreements concerning data extraction will be resolved through discussion. A third reviewer will be consulted where a consensus cannot be reached” |
| Data items | 12 | List and define all variables for which data will be sought (such as PICO items, funding sources), any pre-planned data assumptions and simplifications  Yes- “Extracted data will include: country of origin, study design (parallel/cross-over/cluster, randomisation, allocation concealment & blinding) and professional discipline of clinician delivering the intervention; setting, number of participants per group and points estimates; comorbidities, exclusion/inclusion criteria, participants’ age and gender; type and side of amputation, adverse effects, pain condition and period (months) post-amputation; assessment tools, type of treatment and control intervention received; duration of treatment (minutes), frequency of treatment per week, follow-up period (weeks) and number of patients lost to follow-up; baseline, post-intervention and follow-up results on outcome measures and author conflict of interest statement” |
| Outcomes and prioritization | 13 | List and define all outcomes for which data will be sought, including prioritization of main and additional outcomes, with rationale  Yes- please refer to “outcome measures’ |
| Risk of bias in individual studies | 14 | Describe anticipated methods for assessing risk of bias of individual studies, including whether this will be done at the outcome or study level, or both; state how this information will be used in data synthesis  Yes- Two independent reviewers will utilize the Cochrane method for risk of bias assessment. Included studies will be classified as low, high or unclear risk of bias. |
| Data synthesis | 15a | Describe criteria under which study data will be quantitatively synthesised  Yes- |
|  | 15b | If data are appropriate for quantitative synthesis, describe planned summary measures, methods of handling data and methods of combining data from studies, including any planned exploration of consistency (such as I^2^, Kendall’s τ)  Yes “Methodological heterogeneity will be examined visually, whereas statistical heterogeneity will be assessed through I^2^ statistics. We will consider a cut-off score of 50%. We will use the random effects model to analyse studies with high heterogeneity” |
|  | 15c | Describe any proposed additional analyses (such as sensitivity or subgroup analyses, meta-regression)  Yes- A subgroup analysis depending on age, gender or type of amputee will be performed when applicable.  If numerous trials satisfy the inclusion criteria for this systematic review, sensitivity analysis will be conducted to examine the possibility of excluding studies with a high risk of bias.” |
|  | 15d | If quantitative synthesis is not appropriate, describe the type of summary planned  Yes- a narrative analysis of data will be conducted |
| Meta-bias(es) | 16 | Specify any planned assessment of meta-bias(es) (such as publication bias across studies, selective reporting within studies) |
|  |  | Yes- We will compare the methods section of the articles with the results section. If sufficient data is included, we will assess reporting bias by using a funnel plot (Sterne 2008). |
| Confidence in cumulative evidence | 17 | Describe how the strength of the body of evidence will be assessed (such as GRADE)  Yes- The Grading of Recommendations Assessment, Development and Evaluation(GRADE) |

*** It is strongly recommended that this checklist be read in conjunction with the PRISMA-P Explanation and Elaboration (cite when available) for important clarification on the items. Amendments to a review protocol should be tracked and dated. The copyright for PRISMA-P (including checklist) is held by the PRISMA-P Group and is distributed under a Creative Commons Attribution Licence 4.0.**

*From: Shamseer L, Moher D, Clarke M, Ghersi D, Liberati A, Petticrew M, Shekelle P, Stewart L, PRISMA-P Group. Preferred reporting items for systematic review and meta-analysis protocols (PRISMA-P) 2015: elaboration and explanation. BMJ. 2015 Jan 2;349(jan02 1):g7647.*
